# Soil–Plant Indices Help Explain Legume Response to Crop Rotation in a Semiarid Environment

**DOI:** 10.3389/fpls.2018.01488

**Published:** 2018-11-22

**Authors:** Junxian Li, Kui Liu, Jun Zhang, Lidong Huang, Jeffrey A. Coulter, Trevor Woodburn, Lingling Li, Yantai Gan

**Affiliations:** ^1^Gansu Provincial Key Lab of Arid Land Crop Science, College of Agronomy, Gansu Agricultural University, Lanzhou, China; ^2^Swift Current Research and Development Centre, Agriculture and Agri-Food Canada, Swift Current, SK, Canada; ^3^College of Science, Inner Mongolia Agricultural University, Hohhot, China; ^4^Department of Agriculture Resources and Environment, Nanjing University of Information Science and Technology, Nanjing, China; ^5^Department of Agronomy and Plant Genetics, University of Minnesota, St. Paul, MN, United States; ^6^Faculty of Science, Department of Microbiology and Biochemistry, University of Victoria, Victoria, BC, Canada

**Keywords:** cropping system, legumes, sustainable agriculture, diversification, biotic stress

## Abstract

Crop productivity is typically affected by various soil–plant factors systematically as they influence plant photosynthesis, soil fertility, and root systems. However, little is known about how the productivity of legumes is related to crop rotation systems. The objectives of this study were to determine the effect of rotation systems on legume productivity and the relationships among legume productivity and soil–plant factors. Three annual legumes – chickpea (*Cicer arietinum* L.), pea (*Pisum sativum* L.), and lentil (*Lens culinaris* Medikus), were included in various diversified rotation systems and compared with legume monoculture in the 8-year rotation study. Soil N and water conditions, and canopy and root systems were evaluated at the end of 8-year rotation in the semiarid Canadian prairies. Results showed that diversified rotation systems improved leaf greenness by 4%, shoot biomass by 25%, nodule biomass by 44%, and seed yield by 95% for chickpea and pea, but such effects were not found for lentil. Pea monocultures increased root rot severity by threefold compared with diversified rotations, and chickpea monoculture increased shoot rot severity by 23%, root rot severity by 96% and nodule damage by 219%. However, all the legume monocultures improved soil N accumulation by an average 38% compared to diversified systems. Pea and chickpea displayed considerable sensitivity to plant biotic stresses, whereas lentil productivity had a larger dependence on initial soil N content. The 8-year study concludes that the rotational effect on legume productivity varies with legume species, the frequency of a legume appearing in the rotation, and the integration of relevant soil and plant indices.

## Introduction

The development of sustainable agriculture addresses the dual improvements of crop productivity and soil quality to satisfy the ever-growing food demand driven by the growing human population in the coming 50 years (Tilman et al., 2011; Mueller et al., 2012). However, conventional agricultural development has been heavily dependent on external chemical fertilizers, which contribute to soil degradation, increased costs of crop production, and environmental deterioration (Fedoroff et al., 2010; Malhi et al., 2013). Consequently, reliance on chemical fertilizers is not a solution for sustainable development (Vitousek et al., 2009; Fedoroff et al., 2010). The exploitation of biological N_2_-fixation (BNF) through symbiosis and enhancement of soil nutrient cycling are increasingly necessary to supplement chemical fertilizers in crop production (Peoples et al., 2009; Hossain et al., 2016). Crop rotation is considered as an dual solution to improve soil quality (Wu et al., 2003; Ni et al., 2004; Zotarelli et al., 2007) and crop productivity (Mohr et al., 2011; Gan et al., 2015) through suppressing pests (Gurr et al., 2003) and avoiding pathogen infection (Zhu et al., 2000). Legume-based cropping systems not only increase grain yields, but also improve soil fertility through BNF of legume plants (Siddique et al., 2012). Therefore, increasing legume productivity through optimization of agronomic practices is key to maximize the benefits of legumes in cropping (Gan et al., 2015). Soil N and water conditions are essential factors affecting crop growth, which need to be synchronized spatially and temporally (Zhang et al., 2010). Crop canopy has the dominant role of absorbing solar radiation and CO_2_ necessary for photosynthesis, N_2_ fixation, and carbon sequestration. As a key zone of interaction between legumes and soils (Baron et al., 2013; Oikawa and Ainsworth, 2016), legume root–nodule systems play vital roles in soil nutrient and water acquisition, and symbiotic N_2_ fixation (Carranca et al., 2015). Strategies to synchrony soil nutrient supply and crop demand (Chen et al., 2011; Zhang et al., 2011) are of importance for enhancing N use efficiency and crop productivity (Siddique et al., 2012; Carranca et al., 2015). The growth of legumes is influenced simultaneously by various factors including soil fertility, photosynthesis and root system. The improvement of legume growth and productivity therefore largely depends on the integration of various soil and plant factors (Tittonell et al., 2010; Chen et al., 2011).

Legume-based rotations have been shown to be an effective strategy for improving soil N and water use efficiency (WUE) (Gan et al., 2015), minimizing plant diseases (Holm et al., 2006; Govaerts et al., 2007; Kutcher et al., 2011) and other pest infestation (Govaerts et al., 2007; Kathiresan, 2007), and improving the productivity of subsequent cereals (Davis et al., 2012; Kremen and Miles, 2012; Borrell et al., 2017). However, there are two knowledge gaps specific to legume-based rotation cropping. First, most conventional legume-based rotations have focused on yield response but there are ambiguities when viewed from an integrated soil–crop perspective, how the yield response varies with soil N and water conditions, and crop canopy and root system. Second, although some reports have shed light on how integrated legume rotations can lead to improvement in soil quality, plant growth, and seed yield for the succeeding cereal in the rotation (Chen et al., 2011; Zhang et al., 2011; Gan et al., 2015), there is a deficiency in the understanding how the outcome of crop rotational effect varies with legume species. There is an increasing urgency to systematically determine legume productivity in response to crop species and rotation systems.

Pea (*Pisum sativum* L.), chickpea (*Cicer arietinum* L.), and lentil (*Lens culinaris* Medikus) are the most abundantly-grown annual legumes on the Canadian prairie, where these legumes can fix N_2_ from the atmosphere averaging 54, 52, and 49 kg N ha^-1^ year^-1^, respectively (Lupwayi and Kennedy, 2007; Hossain et al., 2016). The amounts of N_2_ fixed can vary largely, depending on climatic conditions, soil N and water availabilities, and cropping practices (Hossain et al., 2016, 2018). Often, legumes suffer from various biotic pressures (Goodwin, 2008); they are susceptible to pathogenic organisms and diseases, such as ascochyta blight caused by *Ascochyta rabiei* in chickpea (Gan et al., 2006), anthracnose caused by ascomycete pathogen *Colletotrichum lentis* in lentil (Banniza et al., 2018), and root rot complex caused by *Aphanomyces euteiches* in pea (Gossen et al., 2016). Also, legume nodulation is often threatened by nematodes and insects, including weevils, cutworms, and wireworms (Goodwin, 2008). These biotic stresses cause significant leaf chlorosis and wilting, reduction in canopy and root function, and yield losses. Pesticides are commonly used to minimize root rot severity and injury from insects. However, repeated applications of a pesticide can result in pathogens and insects developing resistance to it, and also can cause pesticide pollution with negative ecological consequences (Mohapatra and Roy, 2010). Therefore, it is increasingly important to develop rotation systems for integrated biotic stress management in legume production.

In this study, we integrated several factors including soil N and water conditions, indices of biotic stresses, and crop performance of crop rotation. The objectives were to: (i) determine the effect of crop rotation on soil N and water conditions, legume canopy and root characteristics, and the resulted productivity for three annual legumes, and (ii) evaluate the relationship between legume productivity and the key soil- and plant-related variables. We hypothesized that: (i) the responses to rotation systems for soil N and water conditions, and plant parameters vary with legume species, and (ii) there is a close relationship between legume productivity and sensitive factors, such as soil N and water conditions or crop root and canopy parameters.

## Materials and Methods

### Experimental Site and Design

A field experiment was conducted at the Agriculture and Agri-Food Canada Swift Current Research and Development Centre (50°25′ N, 107°44′ W) from 2009 to 2016. During the growing season (May to September) in 2016, there was 285 mm of rainfall (close the long-term mean of 266 mm for 2009–2016), and mean air temperature was 15.2°C (close the long-term mean of 15.0°C for 2009–2016). The soil was an Orthic Brown Chernozem with the following characteristics in the spring of 2009 in the 0- to 15-cm soil layer: 20 kg ha^-1^ organic C, measured using combustion (Gan et al., 2014); 20 kg ha^-1^ of Olsen P, measured using the Olsen’s methods (Olsen and Sommers, 1982); 380 kg ha^-1^ exchangeable K, measured using ammonium-acetate method (Malhi et al., 2003); and pH of 6.5, measured using an electronic pH meter in a 0.01 M CaCl_2_ solution (PHB-600R, OMEGA Engineering, Canada) (Gan et al., 2015). The experiment included three legumes – chickpea (C), lentil (L) and dry pea (P), each with three levels of rotation diversity: legume monoculture pea-pea-pea (PPP), lentil-lentil-lentil (LLL), and chickpea-chickpea-chickpea (CCC); moderately-diversified rotation systems pea-wheat-pea (PWP), lentil-wheat-lentil (LWL) and chickpea-wheat-chickpea (CWC); and diversified rotation systems of lentil-chickpea-pea (LCP), pea-wheat-lentil (PWL), and lentil-wheat-chickpea (LWC). Each of these 3-year rotations was temporally replicated for two cycles: the first cycle started in 2010 and ended in 2012, and the second cycle continued in the original plots from 2014 to 2016. Spring wheat was planted in 2009 to start the first cycle and in 2013 to start the second cycle; the 2009 wheat was to create a uniform soil condition and the 2013 wheat was to provide a ‘break’ between the legume cycles. Crops in 2016 (i.e., Year 8 in the rotation) were the test crops in which rotational effects are expected to show; thus, the two 3-year rotation cycles are considered together as part of a longer rotation. For example, the PPP treatment was an 8-year rotation of wheat (2009) – pea (2010) – pea (2011) – pea (2012) – wheat (2013) – pea (2014) – pea (2015) – pea (2016). The 2012 chickpea was the third year in the CCC rotation of the first cycle which totally failed due to severe ascochyta blight, thus in the second cycle the Year-2 chickpea in the CCC was replaced with mustard (*B. juncea*), which became CMC.

All crop sequences were arranged using a randomized complete block design with four replications. The plot sizes were 4 m × 12 m with a row spacing of 0.15 m. The cultivars used in the experiment were Brigade for durum wheat, CDC Meadow for field pea, CDC Frontier for chickpea, CDC Maxim CL for lentil, and Cutlass for oriental mustard. These were the most abundantly-grown cultivars in the local area during the years of this experiment (Gan et al., 2009; Niu et al., 2017).

### Crop Management

Crop management practices were consistent across years. Legume seeds were inoculated with *Rhizobium* inoculant at seeding, and all plots were directly-seeded into previous standing stubble using a no-till drill. Pea, chickpea, and lentil were seeded at 1,000,000, 600,000, and 1,500,000 pure live seeds ha^-1^, respectively. Excess N fertilizer application is unbeneficial for legumes to fix N_2_ from air and for soil fertility and quality (Xie et al., 2015; Yong et al., 2018). During the 8-year study, no N-fertilizer was applied to the legumes except for the small portion of N (4.7 kg N ha^-1^) derived from the fertilizer 11-51-0 (N-P_2_O_5_-K_2_O) broadcasted at the rate of 43 kg ha^-1^ to all crops. Wheat and mustard were fertilized with 109 kg ha^-1^ of 46-0-0 and 43 kg ha^-1^ of 11-51-0 (N-P_2_O_5_-K_2_O) at seeding. Thus, 4.7 kg N ha^-1^ and 21.9 kg P_2_O_5_ ha^-1^ were applied for all legumes in 2016. Weeds seized soil nutrients and reduced competitiveness of legume plants, resulting in a weakened or lack of resistance to the pathogen (Whish et al., 2002; Paolini et al., 2006). Weeds and plant diseases were managed using ‘best management practices’ adapted to the local areas. Typically, weeds were controlled using a pre-seeding ‘burn-off’ treatment with glyphosate [*N*-(phosphonomethyl) glycine], and in-crop herbicide application whenever necessary. Ascochyta blight in chickpea and anthracnose in lentil were controlled using foliar-applied fungicides according to recommendations from Agriculture and Agri-Food Canada (AAFC) (Goodwin, 2008; Gan et al., 2015). Legume seed yield was determined by harvesting the central six rows of plants in an area of 14.4 m^2^ (1.2 m × 12 m) in each plot using a combine harvester at full maturity when seed moisture content ≤ 10%. During combine harvest, a 15 cm height of crop stubble was retained in the field, and the remaining straw was chopped by the combine and spread on the soil surface evenly, a common practice used in the local area under no-till management systems (Gan et al., 2014, 2015).

### Soil Sampling and Data Calculation

In each year, soil samples were collected to a depth of 1.2 m within 3 days prior to sowing and again immediately after crop harvest in each plot using a 30-mm diameter hydraulically-driven soil auger. Two soil cores were taken per plot, and each core was divided into 0- to 15-, 15- to 30-, 30- to 60-, 60- to 90-, and 90- to 120-cm increments, and sealed in soil containers for analysis later. Total N was measured from air-dried soil samples using the Kjeldahl N digestion method (Kjeldahl, 1883), and soil water content was measured using the oven-dry method at 105°C (Blake and Hartge, 1986; Fan et al., 2013). WUE and nitrogen use efficiency (NUE) were calculated following the published methods (Sinclair et al., 1984; Raun and Johnson, 1999; Gan et al., 2015), as follow:

WUE = seed mass/(soil water content in the 0- to 120-cm soil layer at seeding – soil water content in the 0- to 120-cm soil layer at harvest + precipitation during the growing season);

NUE = seed mass/(soil N content in the 0- to 120-cm soil layer at seeding – soil N content in the 0- to 120-cm soil layer at harvest + fertilizer N applied to the crop during the growing season).

Water consumption during the growing season was calculated as the difference in soil water content in the 0- to 120-cm soil layer between the sowing and harvest sampling dates plus the precipitation during the growing season. Water loss through drainage and evaporation was none or marginal at the experimental site and thus negligible (De Jong et al., 2008). Nitrogen consumption during the growing season was calculated as the difference in soil N content between the sowing and harvest sampling dates plus fertilizer application. Potential losses of N through leaching is none or marginal at the experimental site and thus negligible (Campbell et al., 2006).

### Canopy Light Interception and Leaf Chlorophyll

At mid-flowering in 2016, photosynthetic photon flux density (PPFD) of diffuse light penetrating through the canopy was determined using a portable canopy light meter sensor (LI-250A, Li-COR, United States). In each plot, the instrument was placed horizontally within the canopy and six positional readings in different positions were taken between 11:00 and 13:00 on a clear day. The average value of the six readings per plot was used for statistical analysis. Six readings outside of each plot without shelter were also recorded as a reference. Five representative plants were marked in each plot at mid-flowering and five largest leaflets of each plant were identified and relative chlorophyll content was measured using a portable chlorophyll meter (SPAD-502 Plus, Konica Minolta, Japan). The severity of shoot rot was determined in each plot at mid-flowering stage using the Horsfall–Barratt scale in 2016, with the severity rated as 0, 1, 2, 3 and 4, representing the percentage criterion of 0–20, 20–40, 40–60, 60–80, and 80–100% of infected leaf area, respectively (Zhu et al., 2000; Gan et al., 2007). Legume shoots were hand-cut at the soil surface at mid-flowering stage from an area of 0.5 m^2^ in the middle of each plot in 2016, and seed and straw were oven-dried at 60°C to a constant weight for biomass.

### Root System Measurement

**FIGURE 1:**
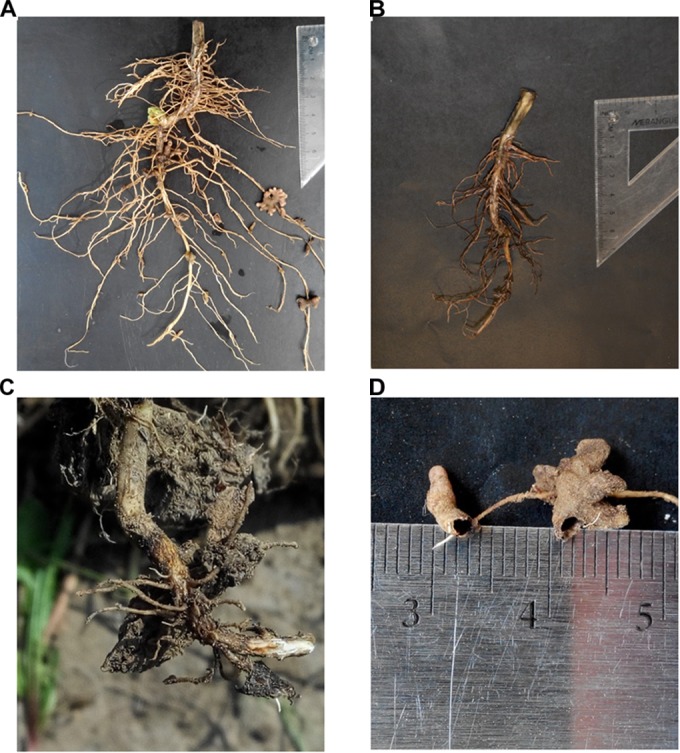
Healthy chickpea root **(A)**, chickpea root with root rot **(B)**, enlarged view of root rot **(C)**, and nodules damaged by insects **(D)**.

Twenty legume plant-root-soil matrixes were dug from each plot to a soil depth of 50 cm at the mid-flowering stage, when the legumes were considered to have the most active nodules (Gan and Liang, 2010). After plant shoots was removed, root–soil matrixes were soaked in water for 24 h at 4°C to remove rhizosphere soil while retaining the nodules on the roots. Those nodules with an internal pink color were considered effective N_2_-fixing nodules (Singh and Varma, 2017). Observations were recorded for the roots that were infected by root rot pathogens (Figures 1A–C) and the nodules that were damaged by nematode or insect (Figure 1D). For each of the sampled plants, the severities of shoot rot and root rot, and nodule damage were assessed using the Horsfall–Barratt scale (Zhu et al., 2000; Gan et al., 2007). All nodules with pink color were removed from roots, and the roots and nodules were oven-dried separately at 60°C to a constant weight and weighed for biomass.

### Statistical Analysis

Data were subject to ANOVA using “lme4” package in R statistical software (version 3.3.1). Normality and variance homogeneity checks were performed according to Shapiro–Wilk’s and Barlett tests at α = 0.05 prior to ANOVA (Zhang et al., 2018). Tukey’s honestly significant difference test (α = 0.05) was used to determine significant differences between rotation systems. Seed yield and shoot to (root + nodule) ratio were normalized by means of calculating relative yield for each of the three legumes; this enabled the determination of rotation systems on each of the legume species included. Correlation analysis was conducted to determine the relationships between seed yield and soil N, soil water, PPFD, leaf relative chlorophyll (SPAD value), shoot rot severity, root rot severity, nodule damage and root biomass. Correlation analysis was also used to determine the relationships between normalized seed yield and normalized shoot to (root + nodule) ratio (Singh et al., 2018). In each of the replicated plots, soil N and soil water content were measured at each of the five soil layers, allowing the determination of the effect of rotation systems on the two soil traits across the entire root-zone profile (Wang et al., 2012) as well as each layer (Liu et al., 2011). The values of total N and water in the 0–120 cm depth were used in correlation analysis. The effect of rotation systems on plant- or soil-related variables was evaluated at the end of the two rotation cycles (i.e., Year-8 of the rotation) to avoid potential confounding factors from variable weather conditions between the first 3-year and the second 3-year cycles.

## Results

### Soil Nitrogen Content

**FIGURE 2:**
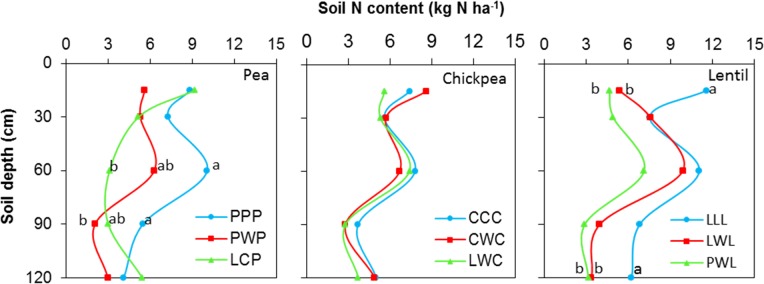
Soil N content in the 0- to 120- cm soil profile at seeding by legume and crop rotation system. Rotation names are detailed in Table 1. Letters denote the significant differences of soil N content in a same layer among treatments, and the soil layers without letter denotes represent no significance among rotations.

**FIGURE 3:**
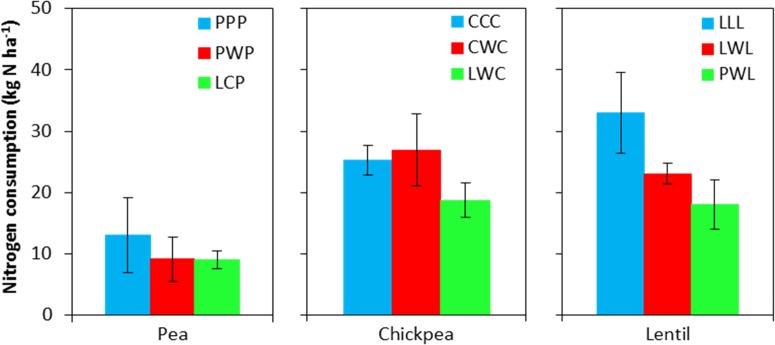
Nitrogen consumption during the growing season. Nitrogen consumption = spring seeding N – fall harvest N + N fertilizer input. Error bars represent one standard error. Rotation names and denotes of statistical significance are detailed in Table 1.

Soil N content in the 0- to 120-cm soil profile responded differently to both legume species and legume frequency in rotations. Residual soil N in the less-intensified pea rotations LCP and PWP were lower by 38 and 61% compared to the monoculture PPP rotation, and displayed significant differences in the 60- to 90-cm soil layer (Figure 2). Residual soil N in the 0- to 120-cm soil layer with the diversified lentil rotation PWL was 43% less than that with the LLL system. Within the 0- to 120-cm soil profile, significant differences were present for the 0- to 15-cm and 90- to 120-cm soil layers for lentil. Although there was no significant N difference in residual soil N among chickpea rotation systems, the residual soil N accumulated in the LWC and CWC rotations was still numerically lower by 19 and 3% than in CCC, respectively (Figure 2). Lentil and chickpea consumed more soil N in growing season compared to pea (Figure 3). For N consumption of each legume species, there was no significant difference among rotation systems.

### Soil Water Content

**FIGURE 4:**
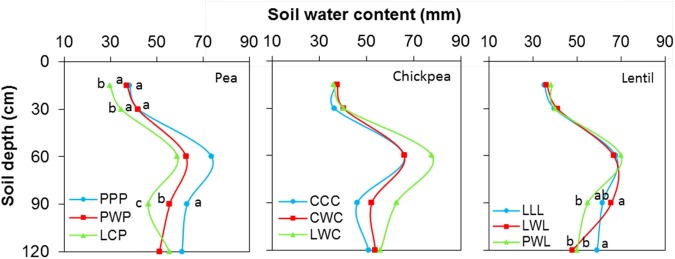
Soil water content in the 0- to 120- cm soil profile at seeding by legume and crop rotation system. Rotation names are detailed in Table 1. Letters denote the significant differences of soil water content in a same layer among treatments, and the soil layer without letter denotes represent no significance among rotations.

**FIGURE 5:**
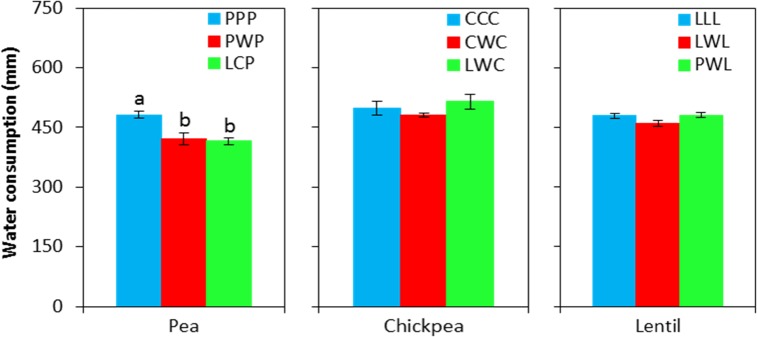
Water consumption during the growing season by legume and crop rotation system. Water consumption = spring seeding water content – fall harvest water content + precipitation in growing season. Error bars represent one standard error, and Rotation names and denotes of statistical significance are detailed in Table 1.

Soil water content was not affected by chickpea-based crop rotations, but significantly affected by pea- and lentil-based crop rotations (Figure 4). For pea-based crop rotations, soil water content in the 0- to 120-cm soil layer for the diversified LCP rotation and moderately-diversified PWP rotation were lower by 23 and 12% compared to the monoculture PPP system, with significant differences in the 0- to 30-cm and 60- to 90-cm soil layers. For lentil-based crop rotations, significant difference in soil water content was displayed in the 90- to 120-cm soil layer. Across all soil layers, the soil water content in the diversified lentil rotation systems PWL and LWL were lower by 2 and 4% than that left in monoculture LLL, respectively (Figure 4). Total water consumption was not affected by crop rotation in lentil or chickpea but was affected in pea (Figure 5). Total water consumption in the 0- to 120-cm soil layer was reduced by 14 and 16% in the diversified LCP and less-diversified PWP than in the pea monoculture PPP, respectively (Figure 5).

### Relative Leaf Chlorophyll and Canopy Size

SPAD value represents the current relative leaf chlorophyll per unit area of leaf (Uddling et al., 2007). Relative leaf chlorophyll content of chickpea was highest with 61.3 SPAD value, and lentil was the lowest with 34.1 SPAD value (Table 1). The less-intensified LCP and PWP systems improved the relative leaf chlorophyll content of pea by 14 and 10% in comparison with the monoculture PPP system. The diversified rotation system LWC improved relative leaf chlorophyll content of chickpea by 13% compared to the monoculture CCC. In comparison, the relative leaf chlorophyll content of lentil grown in diversified PWL was numerically less by 4% than that grown in monoculture LLL (Table 1).

**Table 1:** Relative leaf chlorophyll and photosynthetic photon flux density filtered through the canopy (PPFD) at the mid-flowering stage by legume and crop rotation system.

Legume	Rotation^†^	Relative leaf chlorophyll^‡^ (SPAD value)	PPFD^¶^ (μmol m^-2^ s^-1^)
Pea	PPP	38.5^b§^	142.2^a^
	PWP	42.2^a^	121.4^a^
	LCP	44.1^a^	115.2^a^
Chickpea	CCC	58.3^b^	330.3^a^
	CWC	59.7^b^	293.2^a^
	LWC	66^a^	115.9^b^
Lentil	LLL	34.6^a^	33.7^a^
	LWL	34.4^a^	44.9^a^
	PWL	33.2^a^	43.2^a^


*^†^The rotational sequences were designed using chickpea (C), spring wheat (W), lentil (L), pea (P), and/or mustard (M). Each of these short rotations was temporally replicated for two cycles: the first cycle started in 2010 and ended in 2012, and the second cycle continued in the original plots from 2014 to 2016. Spring wheat was planted to balance the soil condition in 2009 and 2013. The legumes arranged in 2016 were selected as the test crops, thus two cycles of short rotations can be considered as a longer rotation. For example, the rotation PPP was the logogram of pea monoculture which was designed using wheat – pea – pea – pea – wheat – pea – pea – pea. The chickpea in 2015 in CCC monoculture was modified to mustard, because of severe diseases resulted from the chickpea monoculture in the first cycle.*

*^‡^SPAD (soil–plant analysis development) value measured using a SPAD meter.*

*^¶^The reference PPFD without canopy cover averaged 900 μmol m**^-^**^2^ s**^-^**^1^ from 11:00 to 13:00 under clear skies.*

*^§^The letters denote the significant differences of the means among treatments in each variable.*

Photosynthetic photon flux density is an index of canopy size (Table 1). The PPFD of the chickpea canopy in diversified LWC chickpea rotation system was 2.5- and 2.8-fold less than that of the moderately-diversified CWC rotation system and monoculture CCC, indicating that the chickpea in the LWC rotation had the largest canopy size. PPFD of the pea canopy in diversified LCP system was also numerically less than that measured in monoculture PPP. In contrast, lentil in diversified PWL presented a numerically higher PPFD than that measured in the monoculture LLL, suggesting the canopy size of lentil grown in the diversified rotation was inversely smaller.

### Biomass of Shoot and Root Systems

Diversified rotation systems improved shoot biomass of pea and chickpea, especially for chickpea which had 78% greater biomass with the LWC rotation compared to the monoculture rotation, while for lentil which had numerical greater biomass with LLL compared to PWL (Table 2). Rotation system also affected the root–nodule system for the three legumes. The diversified LCP rotation improved root biomass of pea by 44% compared to the monoculture PPP system, and the diversified LWC rotation improved root biomass of chickpea by 87% compare with the less-diversified CWC and CCC systems. The diversified LWC system improved chickpea nodule biomass by 1.6-fold compared with the less-diversified CWC and CCC systems. Thus, diversified LWC improved the total biomass of the root + nodule system by 87%. In contrast, the diversified PWL reduced the biomass of root and root + nodule numerically compared with LWL and LLL. Overall, diversified rotation systems reduced the shoot to root and shoot to (root + nodule) ratios for pea and chickpea but improved the shoot to (root + nodule) ratio of lentil (Table 2).

**Table 2:** Biomass of legume shoot, root, nodule, and root + nodule, along with the shoot/root and shoot/(root + nodule) ratio by legume and crop rotation system.

Legume	Rotation	Biomass (kg DM ha^-1^)				Biomass-based ratio^‡^	
			
		Shoot^†^	Root	Nodule	Root + nodule	Shoot/root	Shoot/(root + nodule)
Pea	PPP	4,366^a^	428.4^b^	85.2^a^	513.6^a^	10.2	8.5
	PWP	4,545^a^	537.2^ab^	115.0^a^	652.2^a^	8.5	7
	LCP	4,970^a^	615.3^a^	73.7^a^	689^a^	8.1	7.2
Chickpea	CCC	2,468^b^	260.3^b^	12.7^b^	273^b^	9.5	9
	CWC	3,716^ab^	404.7^b^	17.0^b^	421.7^b^	9.2	8.8
	LWC	4,393^a^	758.1^a^	43.5^a^	801.6^a^	5.8	5.5
Lentil	LLL	3,914^a^	500.7^a^	7.5^a^	508.2^a^	7.8	7.7
	LWL	3,631^a^	393.3^a^	10.7^a^	404^a^	9.2	9
	PWL	3,746^a^	419.7^a^	6.6^a^	426.3^a^	8.9	8.8


*^†^Shoot biomass excluded seeds.*

*^‡^Biomass-based ratios were calculated based on mean value of shoot, root, and nodule. Rotation names and denotes of statistical significance are detailed in Table 1.*

### Plant Rots and Nodule Damage

All legumes plants were affected by pathogens and nodule insect damage (Table 3). In the local fields, the severity of plant (including shoot and root parts) rot or nodule damage below the threshold of 15% representing a low level. Diversified rotation systems alleviated plant rot and nodulation damage for all legumes. The diversified LCP rotation reduced shoot rot, root rot and nodule damage by 33, 100, and 238% compared to the monoculture PPP, respectively. Chickpea in the diversified LWC had 7.4- and 3.8-fold less root rot compared with the less diversified CCC and CWC systems, respectively. Nodule damage of chickpea in the LWC rotation was 4.4-fold less compared to the CCC system. Lentils in three rotations were influenced by plant rot and nodule insects at a lower level (<15%) compared with pea and chickpea, and these pressures were also numerically lower in the diversified PWL rotation (Table 3).

**Table 3:** Severity of shoot, root rot, and nodule damage by legume and crop rotation system.

Legume	Rotation	Severity (%)		
		
		Shoot rot^†^	Root rot	Nodule damage
Pea	PPP	18.8^a^	20^a^	14.2^a^
	PWP	14.1^a^	10^b^	12.1^a^
	LCP	11.7^a^	6.3^b^	4.2^a^
Chickpea	CCC	88.3^a^	65.4^a^	48.3^a^
	CWC	78.9^ab^	33.3^b^	22.1^b^
	LWC	71.9^b^	8.3^c^	11.3^b^
Lentil	LLL	12.5^a^	10^a^	7.5^a^
	LWL	12.5^a^	4.6^a^	2.9^a^
	PWL	9.4^a^	2.9^a^	2.5^a^


*^†^The plant rots and nodule damage severity < 15% represent a lower level in the local field. Rotation names and denotes of statistical significance are detailed in Table 1.*

### Legume Seed Yield and Nitrogen Use Efficiency

The diversified LWC rotation enhanced seed yield, WUE and NUE by 95, 115, and 165% compared to the less-diversified CWC and CCC systems in 2016, respectively (Table 4). The diversified LCP had numerical improvements for pea’s seed yield, WUE and NUE compared with less-diversified PWP and PPP systems. However, lentil yield and WUE with the monoculture LLL were 17 and 11% greater than those in the LWL and PWL rotations. Significantly greater seed yields of legumes in LCP, LWC, and LLL were also presented in the first cycle (2012).

**Table 4:** Legume seed yield, WUE and NUE by legume and crop rotation system.

Legume	Rotation	Yield (kg seeds ha^-1^)	WUE [kg seeds mm^-1^ (soil water + rainfall)]	NUE (kg seeds kg^-1^ N )
Pea	PPP	4,403^a^	9.2^a^	336.3^a^
	PWP	4,518^a^	10.7^a^	494.8^a^
	LCP	4,690^a†^	11.3^a^	520.7^a^
Chickpea	CCC	1,495^b^	3.0^b^	59.2^b^
	CWC	1,513^b^	3.2^b^	56.1^b^
	LWC	2,954^a†^	6.9^a^	157.7^a^
Lentil	LLL	3,897^a†^	8.1^a^	118.0^a^
	LWL	3,345^b^	7.3^b^	148.3^a^
	PWL	3,425^b^	7.1^b^	185.5^a^


*^†^The significantly greater yields in LCP, LWC, and LLL were also found in the first rotation cycle in 2012. Seed yields of three legumes growing in 2012 were: PPP (2,376 b), PWP (2,581 ab), LCP (2,913 a); CCC (901.94 b), CWC (2,045.53 a), LWC (1,982.24 a); and LLL (2,036 a), LWL (1,783 ab), PWL (1,530 b). Rotation names and denotes of statistical significance are detailed in Table 1.*

**FIGURE 6:**
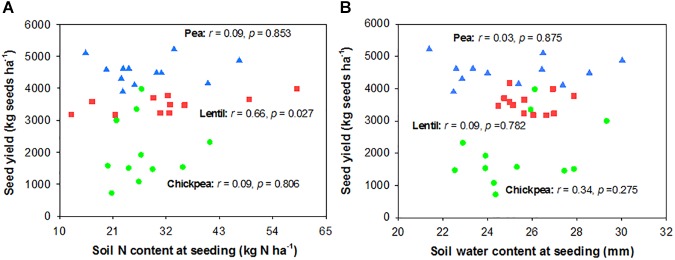
Correlation analysis relating legume seed yields with soil N content **(A)** and soil water content **(B)** at seeding. The red, blue, and green symbols represent lentil, pea, and chickpea, respectively.

**FIGURE 7:**
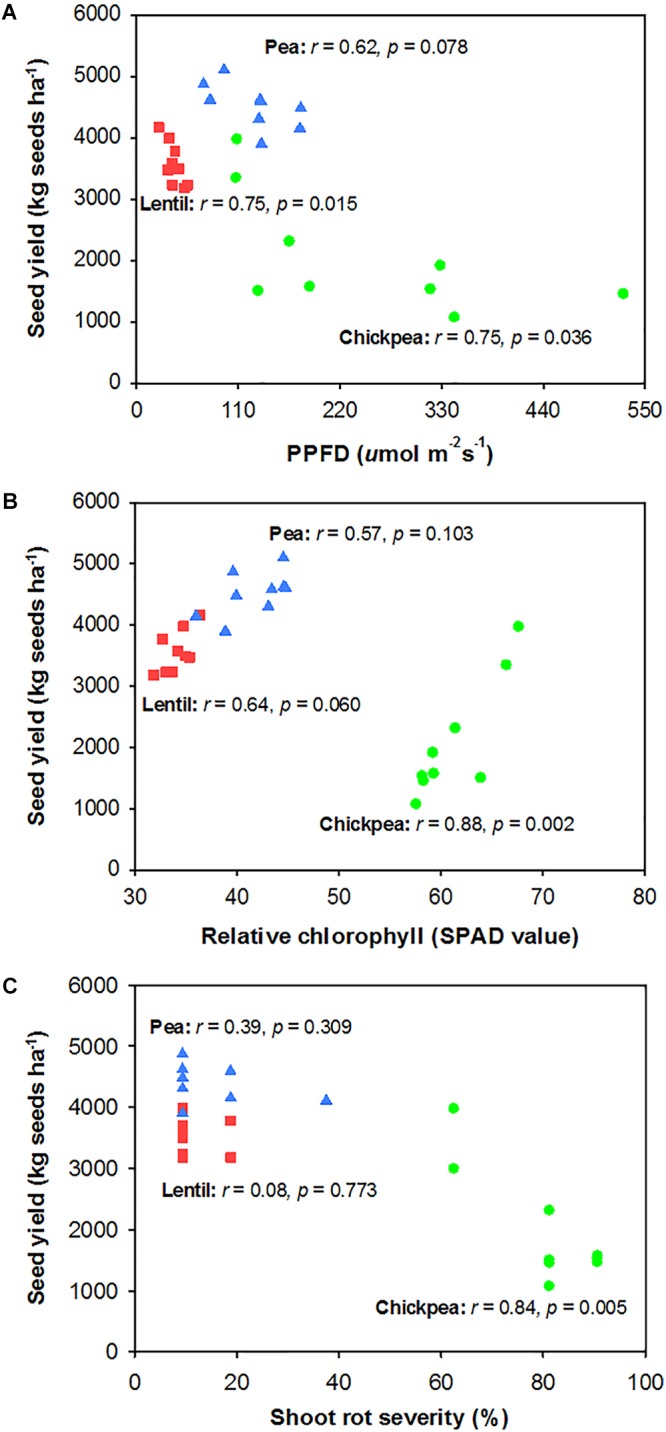
Correlation analysis relating legume seed yield with photosynthetic photon flux density (PPFD) filtered through the canopy (PPFD) **(A)**, relative chlorophyll **(B)**, and shoot rot severity **(C)**. The symbols are detailed in Figure 6.

**FIGURE 8:**
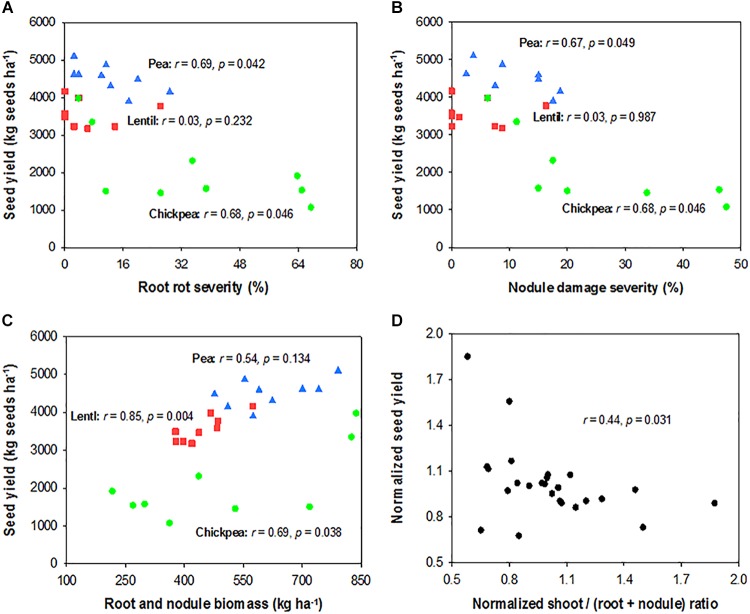
Correlation analysis relating seed yield and root rot severity **(A)**, nodule damage severity **(B)**, and root system biomass **(C)** for each legume, and normalized seed yield and normalized shoot/(root + nodule) ratio for all three legumes **(D)**. The symbols are detailed in Figure 6.

Seed yield of pea and chickpea fields had no significant relationship with soil N content in the 0- to 120-cm soil layer at seeding, while that of lentil was positively related to soil N content at seeding (Figure 6A). Seed yield of legumes had no relationship with soil water content at seeding (Figure 6B). Seed yield of lentil and chickpea had a negative linear relationship with PPFD (Figure 7A). Seed yield of lentil and chickpea had a positive linear relationship with relative leaf chlorophyll content, especially for chickpea (Figure 7B). Seed yield was negatively related to shoot rot severity for chickpea, while there was no significant relationship between these variables for lentil and pea (Figure 7C). Seed yield of pea and chickpea were negatively related to the severity of root rot (Figure 8A) and nodule damage (Figure 8B), while there was no significance for lentil. The seed yield of lentil and chickpea were positively related to biomass of the root + nodule system (Figure 8C). Normalized seed yield was negatively related to normalized shoot/(root + nodule) ratio for all legumes (Figure 8D). In summation, lentil yield was strongly related to soil N content at seeding and was less influenced by biotic stresses. In contrast, yield of pea and chickpea was largely influenced by plant rots, nodule damage, and root + nodule biomass.

## Discussion

Sustainable agriculture addresses the improvement of crop productivity through an integrated ecological approach, such as through the incorporation of disparate biological functions (Fedoroff et al., 2010; Zhang et al., 2011). Diversified cropping systems with legumes rotating with oilseed and cereal have been shown to enhance the systems productivity (Karpenstein-Machan and Stuelpnagel, 2000; Gan et al., 2015), suppress pests (Gurr et al., 2003; Kathiresan, 2007), minimize the development of pathogen resistance (Gan et al., 2006; Kutcher et al., 2011), and enhance environmental sustainability (Gan et al., 2014). These biological functions are achieved through the synthetically improvement of plant growth, soil environment, and plant-soil-microbiome interaction that provides positive feedback to plant growth (Ellouze et al., 2012; Yang et al., 2013; Borrell et al., 2017). Integrated cropping systems approach moves beyond sole perspective to look at the outcome of synergistic effects of the various factors in real practice (Smith et al., 2008; Chen et al., 2011; Coulter et al., 2011). In contrast, monoculture systems often focus on factors from a sole perspective without systematic consideration and the outcome may have a limited value for practical production.

Soil N status and water availability play important roles in regulating the rhizosphere processes which provide feedback to plant growth (Gan et al., 2010; Huang et al., 2016; Borrell et al., 2017). In the present study, the greater soil N content with continuous legumes in a rotation was associated with the return of legume residue to the soil, as legume residue has a relatively low C:N ratio and is easily decomposed compared with cereals and grasses (Johnson et al., 2007; Wang et al., 2015). Greater N consumption with chickpea and lentil compared to pea may have been due to less N_2_ fixation and greater reliance on soil available N (Peoples et al., 2009; Hossain et al., 2016; Rose et al., 2016).

Leaf chlorophyll is an intrinsic factor influencing photosynthesis, and SPAD value is related to chlorophyll content (Uddling et al., 2007; Hu et al., 2014; Bąba et al., 2016). PPFD in crop canopy can be representative of plant canopy size and total leaf area (Oikawa and Ainsworth, 2016), and a crop with a larger canopy can have greater photosynthesis (Radicetti et al., 2012). In our study, legume seed yield was positively related to chlorophyll but was negatively related to PPFD, which was consistent with recent reports by other researchers (Oikawa and Ainsworth, 2016). Our results showed that diversifying crop rotation improved canopy size and leaf chlorophyll status for pea and chickpea, leading to enhanced photosynthesis and productivity, but such an effect was not detected for lentil.

The root performance is reflected by the capacity of roots to penetrate the soil layers for water and nutrient uptake, which is closely related to cropping system (Atkinson, 2000). Greater root mass with diversified crop rotations found in the present study may have been related to less root rot and taxis to N in deeper soil layer, while smaller root mass with monoculture may have been caused by auto-toxicity (Gealy et al., 2013).

Root nodules play an essential role in BNF, and usually larger root nodules are more active for a legume species (Spaink, 2000; Tajima et al., 2008; Singh and Varma, 2017), and roots with more effective nodules usually have greater BNF capacity and resistance to biotic stresses (Yang et al., 2009). Less nodulation in chickpea grown in monoculture in our study may have been related to nutrient simplification or auto-toxicity (Spaink, 2000; Bertin et al., 2003; Bais et al., 2006; Tajima et al., 2008; Gossen et al., 2016). Continuous legume monoculture caused high severities of plant rots in our study, similar to previous findings by others (Govaerts et al., 2007). We also found that the diversified systems decreased root rot severity and minimized nodule damage by insects. Diversifying rotation systems has been reported to buffer biotic stresses successfully (Gan et al., 2006; Govaerts et al., 2007; Kutcher et al., 2011).

Soil N and legume canopy and root systems have a complicated bilateral causality (Tittonell et al., 2010; Chen et al., 2011). First, N in deeper soil layers cannot be absorbed by the plant if the fine roots are not long enough (Liu et al., 2010), which could increase N accumulation in deeper soil layers (Shen et al., 2013). Larger shoot and root systems with less disease in diversified rotation systems stimulated the deep expansion of tap or lateral roots, likely increasing N uptake and contributing to less residual soil N after harvest (Mi et al., 2010; Shen et al., 2013). In the present study, less residual soil N in the diversified LCP and LWC systems was likely the result of larger shoot and root systems. Our correlation analyses revealed that seed yields of pea and chickpea were negatively related to biotic stresses, and there was no relationship between seed yield and soil N content at sowing. These results indicate that pea and chickpea are not sensitive to soil N and their N demands are mainly dependent on biologic fixation. Second, the synchronization between N supply and crop demands is important for the development of plant canopy and root systems (Shen et al., 2013). Root growth and expansion can be greatly constrained when soil N supply is low, particularly during early growth (Peng et al., 2010).

In the present study, lentil yield had no significant linear relationship with biotic stresses but was positively related to soil N content at seeding, indicating that lentil yield is less influenced by biotic pressures compared to soil N content at seeding (Singh et al., 2018). Greater lentil yield in three-continuous-lentils included LLL compared with that in the moderately-diversified LWL and PWL systems was likely related to sufficient fixed-N accumulated in soil with lentil monoculture (Huang et al., 2016). In legume, *Rhizobium* bacteria accumulation in soil due to continuous legume cropping can enhance root nodulation and BNF (Furseth et al., 2012).

Overall, this study showed that legume species responded to crop rotation systems differently in terms of root and nodule formation, BNF, and the capacity to tolerate biotic stresses. Significant positive outcomes in legume productivity derive from the integrated synergy of the selection of legume species, enhancement of soil fertility and growing environment, and reduction of biotic pressure resulted from rotation diversification. A key practice of achieving these positive outcomes is disease management for pea and chickpea and soil N management for lentil. However, our findings from this 8-year rotation study may have substantial limitations, as rotational effects on legume productivity could vary with different cultivars (Bazghaleh et al., 2015), preceding crops in rotation (O’Donovan et al., 2014; St. Luce et al., 2016), soil physiochemical and biological properties (Bainard et al., 2016; Niu et al., 2017), and climatic conditions and testing environments (Hossain et al., 2016),

## Conclusion

The assessments of soil water and N conditions, crop canopy characteristics, root systems and nodule formation, and seed yield revealed that the effects of rotation systems could vary with legume species. Diversified rotation systems significantly improved relative leaf chlorophyll, shoot and root biomass, and seed yield for chickpea and pea, but not for lentil. Continuous legume production increased the severity of shoot and root rots and nodule damage for all legumes. Lentil monoculture had stronger resistance to biotic stresses, leading to higher seed yield than the rotation including fewer lentils. There was no relationship between seed yield and soil N content at seeding for chickpea or pea, but a linear relationship existed for lentil. Continuous legume rotation improved soil N status overall, while the productivity of lentil depended on soil N supply more than those of chickpea and pea. This study highlights the importance of the integrated synergy among the key soil and plant factors sensitive to crop growth for sustainable legume production.

## Author Contributions

YG conceived the idea and designed the project. JL performed the data collection and analysis together with LH and JZ. JL wrote the draft of the manuscript guided by LL and YG. KL, JAC, and TW contributed to the manuscript enhancement. YG finalized the article.

## Conflict of Interest Statement

The authors declare that the research was conducted in the absence of any commercial or financial relationships that could be construed as a potential conflict of interest.
